# Anxiety and depression in older patients: the role of culture and acculturation

**DOI:** 10.1186/s12939-017-0666-z

**Published:** 2017-10-04

**Authors:** Anna Zisberg

**Affiliations:** The Cheryl Spencer Department of Nursing, Faculty of Social Welfare and Health Studies, University of Haifa, Mount Carmel, 31905 Haifa, Israel

**Keywords:** Acculturation, Culture, Older adults, Depression, Anxiety, Hospitalization

## Abstract

**Background:**

Anxiety and depression are major health concerns in general among older adults and especially during hospitalization, as they lead to numerous negative outcomes. There is currently no sufficient body of research examining the role of cultural background in patients’ experience of these conditions. Better identifying patients at risk may help reduce inequity and provide patient-centered, culturally sensitive care. The current study explores the roles of culture and acculturation in anxiety and depression levels in recent and veteran Russian immigrants compared with native Israelis and veteran immigrants from Middle Eastern countries.

**Methods:**

Secondary analysis of a prospective cohort study of cognitively intact older adults (70+) hospitalized for acute conditions in internal medical units in two hospitals in Israel during 2009–11. Depression and anxiety were assessed within 48 h of admission through personal interview using the Tucker Depression Rating and the Short Anxiety Screening Tests. Demographic and health data were collected from electronic health records. Immigration status was defined by country and emigration year. Study hypotheses were tested employing analyses of covariance, modeling anxiety and depression symptoms separately, controlling for potential confounders.

**Results:**

Significant differences between study groups were observed in fully adjusted models for anxiety symptoms (*F*_[3, 515]_ = 5.24, *p* < .01) when both veteran (21 ± 5.83) and recent (20.2 ± 5.23) Russian immigrants expressed higher anxiety levels than native Israelis (18.35 ± 5.23) and veteran immigrants (18 ± 5.03) (from *p* = .05 to *p* < 0.01). No significant differences were found in anxiety symptoms between recent and veteran Russian immigrants. Both depression and anxiety symptoms showed an interaction effect of study immigration groups by sex: while no differences were observed among native Israelis, significantly higher depression and anxiety were observed among women than men in the other groups.

**Conclusions:**

Culture of origin may play a central role in determining expression of anxiety symptoms and perhaps modify acculturation. During hospitalization, special attention should be given to the level of anxiety among not only recent but also veteran immigrants. Further research may explore whether elevated anxiety is a result of stress due to hospitalization or a stable trait.

## Background

Among hospitalized older adults, anxiety and depression can be considered additional risk factors and serious comorbidities [1, 2]. Although both conditions are associated with increased risk of functional decline [3, 4] and decreased adherence to treatment and recovery [5, 6], routine screening for them is not common in hospital settings [7]. It is therefore in the best interest of patients and care providers alike to better understand risk factors associated with depression and anxiety among this unique target population, which is quickly becoming the health system’s prime client.

Anxiety and depression are related conditions that compose a major comorbidity for many health conditions among older adults. Their effect is multisystemic, involving cognitive processes, perception, and comprehension; afflicting motivation, interpersonal communication, and relationships; and, at least according to some of the evidence, undermining physiological functions that exacerbate existing and new health conditions [8, 9]. However, unlike other comorbidities, both conditions are based on subjective reactions of individuals to conditions (internal and external) that they interpret as intimidating and threatening [10]. Therefore, these conditions are influenced more by factors that shape individuals’ perceptions and interpretations of their reality, chief among them culture.

Despite the well-documented association between aspects of culture and individual perceptions and interpretations of diverse life conditions, health conditions included [11], work in this venue is still preliminary: no comprehensive body of research examines the role of cultural background in patients’ experience of depression and anxiety. The current study addresses this lacuna by examining the role of culture and acculturation (length of time living in a host culture) by comparing groups of native older adult patients and immigrants from different cultural groups and varying lengths of stay in a host culture on measures assessing symptoms of anxiety and depression.

### Anxiety and depression as health risks and comorbidities

Anxiety and depression are two conditions sharing core components while maintaining separate meanings in various aspects: anxiety is a multisystemic response to conditions (internal or environmental) that are judged by individuals to be threats or severe challenges to their existing coping resources; such conditions can be related to health, a change in the environment (such as immigration), or ongoing challenges in personal relationships, to name just a few [12]. Unlike stress, anxiety is an ongoing experience that goes beyond the specific need (objective or subjective) to recruit resources and manage a challenge. It has to do with lingering stress and the experience of distress that, if unaddressed, may result in depression.

Depression is a clinical condition that has become more and more prevalent in Western societies in recent years [13]. While not necessarily more common among older adults, the condition is considered potentially more hazardous to older adults, especially those with challenging health conditions [5, 14]. Depression involves an ongoing extremely negative mood, accompanied by negative perceptions of life situations and self, typically resulting in withdrawal from interpersonal relationships and life commitments (like work or childrearing), and in extreme cases may result in behavior aimed at harming self and others [13, 14]. Depression is considered a major condition in its own right, and when accompanying additional health challenges it may undermine adequate coping, the gaining of social and professional support, and adherence to care procedures to facilitate recovery and regain function and well-being.

Anxiety and depression are both health risks and dangerous comorbidities. Both are associated with depletion of personal resources for coping with challenges and thus may either create susceptibility to various health conditions or tax coping resources, thereby reducing the chances of adequate coping and recovery. Both conditions are based on individuals’ perceptions of, interpretations of, and emotional responses to internal and external cues. The evidence is unanimous that subjective perceptions and interpretations of one’s life conditions to some of the major factors triggering anxiety and depression [15]. In this study, I propose that culture, a general frame of reference shaping group and individual perceptions and judgements, may be associated with both conditions.

### Culture and acculturation as a frame of mind and a potential risk factor for anxiety and depression

Culture is a broad term approached from myriad theoretical points of view and disciplines; surprisingly, most of them agree on its core definitions. Culture can be viewed as an amalgam of basic assumptions, norms, and values shared by members of a given social group. These define their perceptions of their reality, how they interpret it, and thus how they react and behave [16, 17]. So powerful is culture as a factor shaping individual perceptions and behavior that empirical evidence has linked cultural background with individual visual memory, emotional reactions to environmental cues, and reactions to emotional stimuli [18, 19]. No wonder, then, that preliminary evidence suggests that culture plays a major role in individuals’ perceptions of and reactions to various health conditions [20].

Individuals are either born into a culture or introduced to it later in life as they move from one cultural setting to another. Modern trends of immigration and globe-trotting put larger and larger portions of the world’s population into the latter category. What happens to an individual or group born into one culture and moved to another? Firstly, immigration itself is often portrayed in the literature as crisis. Although the case examined here focuses on immigrants by choice (i.e. not refugees), the process is still marred with difficulties: physical, formal (i.e.: coping with bureaucratic aspects of immigration), socio economical and emotional, to name just a few levels of conflict and abrupt change accompanying such move [21–23]. In other words: even if all goes well, and formal aspects of immigration and resettlement go as expected (for example – achieving immigrant status legally, learning the local language, gaining education, training or employment), the process can still be defined as a social and psychological crisis, where identities, sense of belonging in a community, social and financial security and social support are often lost and need to be re-negotiated. Immigrants are often pointed out as a high risk population when it comes to health and behavioral patterns reflecting challenged adjustment, chief among them are depression and anxiety [24, 25].

The process of adjusting to a new culture is referred to as acculturation, or the process of change in individual aspects of identity associated with a given culture when it interacts with another one [26]. The term became a pillar of research on the psychosocial changes accompanying immigration, intergroup dynamics, and practically any ongoing meeting of cultures. Studies on immigrants, expatriates in global business settings, and social subgroups coexisting within the same social and political boundaries have exposed how difficult acculturation is and how intricate are the dynamics of gradually adopting a new culture as one’s own while coping with the old culture and its implications for individuals’ and groups’ identities, norms, and behaviors [27]. There is general agreement in the literature that shifting between cultures is stressful and challenging [26, 27].

Acculturation also refers to the extent to which individuals are rooted in their new society and thus, among other things, gaining a social support network, identified in current studies as one of the best antidotes to anxiety and depression [28]. Culture and the process of acculturation among immigrants can play more than one role in shaping anxiety responses and even depression. First, cultures vary in the extent to which they allow emotional expression and in how they shape the expression and communication of emotion [29, 30]. Culture also changes the experience of care and access to available care resources [31, 32]. In these respects, individuals from minority groups, or relatively new immigrants, are less versed in negotiating with the host-culture health system and may experience higher distress in dealing with it or using its resources. Second, acculturation may offer yet additional sources of variability in how well individuals cope with health challenges and with health systems and in how they express their experiences [33].

### The case of Russian immigrants in Israel

What role do cultural background and acculturalization play in the variance in anxiety and depression among older adult patients? This study examined the question using a cross-sectional/comparative design set in the context of an immigrant-absorbing society, the state of Israel, that offers samples of both recently arrived and veteran immigrants in a manner that allows control over socioeconomic- and access-related factors.

Israel has been welcoming immigrants from all over the world since its inception. All legal immigrants are granted, upon arrival, full health insurance coverage; and since Israeli society (much like U.S. society and others) is a society of immigrants, recently arrived patients are likely to find within the health system professionals who speak their native language. These aspects ameliorate language and financial issues that often interfere with individuals’ access to and knowledge of the health system, especially at an older age. The largest share of immigrants into Israel in recent decades originated in the former Federations. About 1.5 million of Israel’s 7.5 million citizens are from Russia or the former Soviet Union [34]. A little less than half arrived in the 1970s, and the rest during 1990–2000. In practical terms, former Soviet Union immigrants could be divided into two clearly demarcated time frames of arrival separated by about 20 to 25 years. This is a suitable condition for testing the potential effects of acculturation on otherwise almost identical groups of immigrant target populations.

Russian culture, though varying across many republics of origin and socioeconomic strata, shares a few core values and assumptions that are crucial to the current investigation. Russian culture is considered by most authors in the field to be a highly emotional one. Positive and negative emotions are experienced and expressed with little (and sometimes no) filters of ‘decorum’. However, expression of emotion is very physical and is often associated with physical displays. In other words, Russians do not talk emotion so much as ‘do’ emotions and express them in physical gestures or acts [35]. Native Israelis, by comparison, are considered to be more openly verbal about emotional experiences, especially negative experiences [36]. Studies examining the characteristics associated with health and wellbeing among this group of immigrants show a relatively complex picture: On one hand, new immigrants from the former Soviet union are characterized by higher human capital (e.g.: higher education); on the other hand, we see differing patterns of social support among immigrant and non-immigrant groups and higher levels of distress and poorer self-reported health in this group [37, 38]. A recent longitudinal study that assessed loneliness and depressive symptoms among new immigrant and veteran immigrants in Israel found that newer immigrants from the former USSR were disadvantaged on all levels compared with veterans, but this gap narrowed along time – probably representing processes of acculturation [39].

Thus the setting of hospital care in Israel provided a suitable natural social setting that enabled the comparison of native older adults with veteran Russian immigrants, recently arrived Russian immigrants, and, as an additional control group, veteran immigrants, mostly from Middle Eastern and Arab cultures of origin. I compare anxiety and depression levels between the groups while controlling for background variables (age, gender, social support, health, and functional status) to test the potential roles of culture and acculturation in their occurrence and level of expression. Based on the evidence reviewed above, I hypothesized the following. (a) The four groups will show differences in levels of anxiety and depression symptoms. (b) Recent immigrants will show the highest levels of symptoms, native Israelis will show the lowest level of symptoms, and, due to the nonverbal emotional display rules of emotions among individuals from Russian cultures, (c) veteran Russian immigrants will display higher levels of symptoms than veteran immigrants from other cultures.

## Methods

### Data and sample

This study analyzed data from the prospective HoPE-FOR (Hospitalization Process Effects on Functional Outcomes and Recovery) study carried out in eight internal medicine units at two tertiary centers in northern Israel between 2009 and 2011. The original study recruited patients 70 and older who were admitted for a non-debilitating condition and who were capable of communicating in one of the three main languages in Israel (Hebrew, Arabic, and Russian). The eligibility criteria, the recruitment process, and attrition are fully described elsewhere [3]. Of the 690 potential participants, I excluded 142 whose reports relied on proxy interview; another 12 (2.2%) were excluded due to missing data on depression and anxiety assessments. The remaining participants (*N* = 536) were grouped by the following criteria: (1) time since immigration (born in Israel, emigrated before 1951 [at age < 12], emigrated between 1951 and 1989, emigrated after 1989), and (2) native-language Russian or not. Four study groups were defined: native Israelis (NIs, *n* = 87); those who emigrated from different countries as children (before 1951), or veteran immigrants (VIs, *n* = 197); those who emigrated from Russia before 1989, or veteran Russian immigrants (VRIs, *n* = 51); and recent Russian immigrants, arriving after 1989 (RRIs, *n* = 197). Study protocols were approved by the two hospitals’ Helsinki committees and the university’s institutional review board.

### Measures

#### Anxiety symptoms

Participants completed an instrument that screens for anxiety symptoms in older adults 48 h after hospital admission. The Short Anxiety Screening Test (SAST) [40] is a 10-item, 4-point Likert-scale questionnaire that conforms to the criteria for anxiety described in the DSM-V. Total scores range from 10 to 40; higher scores represent more severe conditions. The instrument has a sensitivity of 75% and specificity of 79%, with a Cronbach’s alpha of 0.70 [40] and 0.71 in the current study.

#### Depressive symptoms

Depressive symptoms were measured 48 h after hospital admission using the 10-item Short Zung Interviewer-Assisted Depression Rating Scale [41]. Responses were rated on a 4-point scale from 1 (*never*) to 4 (*most of the time*). Total scores are transformed to a 100-point scale and range from 25 to 100; higher scores indicate a greater number and frequency of depressive symptoms. The instrument has been validated as a screening tool for the presence of depressive symptoms in older adults [42]. Reliability for the current study was good (Cronbach’s alpha = 0.72).

#### Control variables

Sociodemographic variables and variables related to functional cognitive and health status were included in the data analysis as potential confounders. *Functional status* on admission was assessed using the 11-item Modified Barthel Index (MBI) [43], consisting of individuals’ self-assessment of their independence in performing basic activities of daily living (ADLs). *Cognitive status* was measured using the Pfeiffer Short Portable Mental Status Questionnaire [44]. Total scores ranged from 0 to 10 correct items; higher scores indicated better cognitive status, and participants who scored below 6 required proxy report and were not included in the current study. *Severity of acute health conditions* was measured with the Acute Physiology and Chronic Health Evaluation (APACHE II) [45], a valid and reliable assessment of severity of illness in acute conditions. The APACHE II uses a point score (range 0–71) based on the initial values of 12 routine physiological measurements, age, and previous health status to provide a general measure of severity of disease. *Severity of chronic health condition* was assessed using Charlson’s comorbidity index, which weights 20 health conditions and their severity on a scale from 1 to 6. Predictive validity of the index was shown to be high, using criteria such as likelihood of death [46]. The last two assessments relied on information retrieved from patients’ electronic medical records. Given the established fluctuation of mental status by *age, gender,* and *social support,* these variables were also included. *Social support* was assessed based on participants’ report of the number of hours (0–12) that they have visitors on an average hospitalization day.

### Data analysis

Means and standard deviations were used to describe continuous variables, and percentages were used to summarize categorical variables. All study variables were presented for the four study groups. Group differences were compared using univariate analyses of variance with Bonferroni adjustment for multiple comparisons for continuous variables and chi-squares for categorical data. To test the main study hypothesis, I employed analyses of covariance (ANCOVAs), modeling anxiety and depression symptoms separately and controlling for the potential confounders functional, cognitive, and health status as well as demographic variables. I chose a conservative approach and used a 0.15 threshold level in the univariate analysis to determine variable inclusion in the multivariate models. Data were analyzed using IBM SPSS Statistics version 23.0.

## Results

### Sample characteristics

The mean age of the participants was 78.3 years (*SD* = 5.7), 48% were female, 85% reported being independent in basic functions on admission (MBI cutoff of 80) [47], 16% met the criteria for depression (score ≥ 70) [41], and 23% met the criteria for anxiety (score ≥ 24). Almost all (94%) participants reported receiving support from family and friends for at least an hour a day, and 50% reported 4 or more visiting hours. Descriptive statistics for all study variables by immigration status are presented in Table 1.

**Table 1:** Descriptive statistics of the sample and main study variables, by study groups (*N* = 536)

Characteristics	Natives (NIs) (*n* = 87)	Veteran immigrants (VIs) (*n* = 197)	Veteran Russian immigrants (VRIs) (*n* = 51)	Recent Russian immigrants (RRIs) (*n* = 197)	*F*/^χ2^
Age, mean ± SD^#^	76.00 ± 4.91	79.81 ± 5.72	79.10 ± 6.38	77.66 ± 5.43	11.02^***^ NI < VI = VRI, VI = VRI > RRI
Gender (female), *n* (%)	29 (33.3)	80 (40.6)	30 (58.8)	115 (58.4)	22.7^***^ VRI = RRI > NI = VI, VRI > NI, VI
Year of immigration, mean ± SD	–	1945 ± 5.29	1969 ± 12.18	1994 ± 4.09	3194.07^***^ VI < VRI < RRI
Social support (hours), mean ± SD	5.68 ± 3.91	5.57 ± 3.57	5.29 ± 3.91	3.18 ± 2.48	21.43^**^ RRI < NI = VRI = VI
Cognitive status, mean ± SD	8.69 ± 1.56	8.70 ± 1.40	8.06 ± 2.08	8.96 ± 1.30	5.17^**^ VRI < RRI, VI, NI = VI = RRI
Functional status (^^^ADL), mean ± SD	91.70 ± 16.08	91.53 ± 17.06	90.71 ± 14.52	94.87 ± 9.99	2.39, *p* = 0.08
Severity of acute illness, mean ± SD	11.81 ± 4.37	11.67 ± 4.73	10.02 ± 3.50	10.84 ± 4.43	1.75, *p* = 0.15
Comorbidity (Charlson), mean ± SD	2.70 ± 2.23	2.68 ± 2.25	2.31 ± 1.91	2.28 ± 2.07	1.66, *p* = 0.14
Cardiovascular diagnosis, *n* (%)	32 (37.2)	59 (29.9)	22 (43.1)	78 (39.6)	5.42, *p* = 0.14
Depression symptoms, mean ± SD	51.88 ± 15.01	51.45 ± 15.08	56.12 ± 16.72	51.09 ± 14.84	1.68, *p* = 1.15
Anxiety symptoms, mean ± SD	18.35 ± 5.23	18 ± 5.03	21 ± 5.83	20.2 ± 5.23	7.42^***^ VRI = RRI > VI = NI, VRI > VI, NI

^*^*^ *ADL* activities of daily living, ^#^ *SD* standard deviation. **p < .01; ***p < .001

### Descriptive and univariate statistics of study variables by immigration status group

Univariate comparisons showed no significant differences for medical conditions (severity of acute and chronic illness, primary diagnosis of cardiovascular illness, and physical function) and depression symptoms only. All other characteristics differed between at least two of the study groups.

Participants in the VRI and RRI groups expressed significantly higher symptoms of anxiety (*F*_[3,528]_ = 7.42, *p* < .001) and included more females (χ^2^ = 22.7, *p* < .05) than the NI and VI groups. RRIs and VIs differed significantly for age, cognitive status, and provided social support: RRIs were on average 2 years younger, had an almost 1 point higher cognitive score, and had 2 less visiting hours. NIs and VIs (non-Russian) were similar across all variables besides age: NIs were on average 3 years younger than VIs. The complete group comparison is shown in Table 1. Given my conservative significant level of ≥0.15 for all study variables, I included all control variables in the multivariate analysis.

### Multivariate analysis

Significant differences between study groups were observed for anxiety symptoms in the fully adjusted model (*F*_[3, 515]_ = 5.24, *p* < .01) but not for depression symptoms, controlling for the same variables (*F*_[3, 515]_ = 0.89, *p* = NS). Bonferroni post-hoc tests performed only for anxiety symptoms revealed that anxiety was significantly higher in the VRI group than in the NI (*p* = 0.05) and VI (*p* = 0.02) groups, and significantly higher in the RRI than in the VI (*p* < 0.01) group. No significant differences were found in anxiety symptoms between recent and veteran Russian immigrants.

**Fig. 1:**
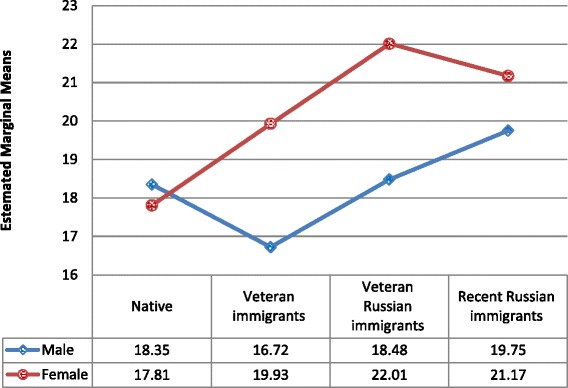
Anxiety symptoms, by immigrant status group and gender

**Fig. 2:**
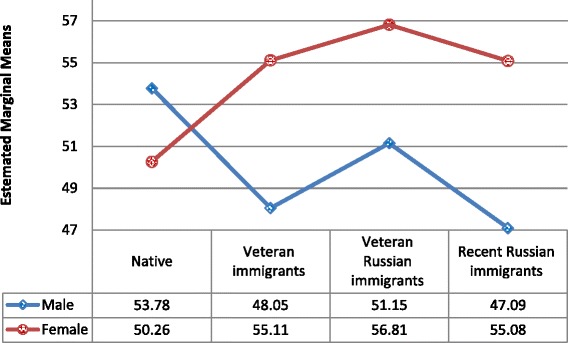
Depression symptoms by immigrant status group and gender

Sex was one of the strongest predictors of both depression and anxiety symptoms and therefore raised concerns about potential interaction in relationships between immigration status and anxiety/depression symptoms. Therefore, an interaction term (Immigration status group × Sex) was entered into the fully adjusted models. As Table 2 shows, interaction terms for both anxiety and depression are significant. And close inspection of Figs. 1 and 2 shows that there is no significant difference in anxiety and depression in the NI group, whereas all other groups followed the same trend of females expressing more symptoms than males.

**Table 2:** Association between immigration status group, depression, and anxiety symptoms and confounders

	Depression symptoms		Anxiety symptoms	
	Model 1 (Adjusted)*F* (df)	Model 2 (Adjusted + Interaction) *F* (df)	Model 1 (Adjusted)*F* (df)	Model 2 (Adjusted + Interaction)*F* (df)
Age	5.44 (1)^*^	6.13 (1)^**^	0.36 (1)	0.20 (1)
Social support (hours)	4.39 (1)^*^	4.69 (1)^*^	0.13 (1)	0.10 (1)
Cognitive status	26.97 (1)^***^	27.42 (1)^***^	6.12 (1)^*^	6.16 (1) ^**^
Functional status (^^^ADL)	12.61(1)^***^	12.16 (1)^***^	16.45 (1)^***^	15.69 (1)^***^
Severity of acute illness	2.30 (1)	2.96 (1)	1.53 (1)	2.16 (1)
Comorbidity (Charlson Index)	1.52 (1)	1.46 (1)	1.37 (1)	1.15 (1)
Cardiovascular diagnosis (Yes)	0.21 (1)	0.32 (1)	1.00 (1)	0.66 (1)
Sex (Female)	19.43 (1)^***^	8.45 (1)^***^	25.32 (1)^***^	12.89 (1)^***^
Immigration status group	0.89 (3)	0.65 (3)	5.24 (3)^***^	5.84 (3)^**^
Immigration status group × Sex	–	3.43 (1)^*^	–	2.76 (1)^*^

^*^*^ *ADL* activities of daily living. *p < .05; **p < .01; ***p < .001

Subsequent analysis (ANCOVA) performed separately for men and women confirmed the above findings, with the same pattern of differences in women (anxiety: *F*_[3, 243]_ = 3.43, *p* < 0.05; depression: *F*_[3, 243]_ = 0.75, *p* = NS). In the subsample of males, anxiety symptoms were significantly different (*F*_[3, 266]_ = 4.59, *p* < 0.05) between immigration groups. For depression, the results were not significant, but a trend was observed in a similar direction (*F*_[3, 266]_ = 2.27, *p* < 0.08). Bonferroni post-hoc tests performed separately for male and female subgroups demonstrated that in males anxiety was significantly higher for the VRI than for the VI (*p* = 0.03) group and for the RRI than for the VI (*p* < 0.03) group. No significant differences were found in anxiety symptoms between recent and veteran Russian immigrants. Also, for NI males, anxiety was not significantly different from anxiety for any other groups. For women, the differences in anxiety between immigration status groups were the same as those observed for the entire sample.

## Discussion

While the literature acknowledges the potential role of culture in providing health care and addressing health care disparities [48], especially in older patients [1, 5], there is very little evidence-supported literature in this venue to feed future model development and practice. The current study provided evidence that partly supported my hypotheses, demonstrating some of the complex ways and routes by which culture and acculturation may affect depression and anxiety as psychosocial comorbidities among older adults in acute hospitalization settings.

My main results demonstrate how patients of different cultural backgrounds show varied levels of anxiety. Anxiety is a major factor in determining not only patient well-being but also hospitalization-related outcomes such as higher risk of falls [4], elevated risk of mortality, rehospitalization, and additional complications and adverse outcomes [49]. This evidence accounts for the importance accorded anxiety in the literature as an outcome in its own right as well as a comorbidity.

While the literature supports a close association between anxiety and depression [50], my results support differences between cultural groups in anxiety symptoms only, with a similar yet nonsignificant trend in depressive symptoms: as hypothesized, immigrants from the former USSR showed higher levels of anxiety than all other groups, including native Israelis and veteran immigrants from other cultures (mostly Middle Eastern). Contrary to my hypotheses, both veteran and recent Russian immigrant groups showed the same result patterns. In addition, despite the fact that new immigrants from the former USSR received less social support than their veteran peers, both groups showed similar (and higher) levels of anxiety. Such results may suggest that culture of origin plays a more central role in patients’ responses than acculturation (time within the new or host culture).

With only partial accordance with my hypotheses, I did not find significant differences between the non-Russian groups (native Israelis and other veteran immigrants) in levels of depression and anxiety. Generally, my results suggest that culture-specific characteristics play a role in anxiety (and to some extent depression) levels among acutely hospitalized older adults, and that acculturation does not affect these groups. How does my finding fit with the ample evidence on the role of acculturation? [51]. One possible answer is respondents’ age: all participants in this study were older adults who had passed all of their formative years within the culture of the former Soviet Union and were hence perhaps less touched and shaped by their new host culture. Existing evidence points to the role of age in acculturation [52]. Thus, my evidence strengthens our knowledge of the centrality and importance of culture of origin among older patients and the ways they cope with and react to health conditions and to care.

Additional findings exceed the scope of my original hypotheses but are worth discussing: patients of Russian origin also tend to suffer higher rates of cardiac complaints and conditions [53, 54] and as a result are at higher risk of cardiovascular disease. Could it be that these specific conditions brought about the more pronounced anxiety symptoms? To address this possibility, I controlled not only for severity of illness but also for the specific complaints typical of these groups. The results reported remained unchanged and significant after controlling for these potential confounders.

Gender was another potential factor of interest, beyond my hypothesized effects. When controlling for gender in all the analyses, potential interactions between gender and the study main factors were weighed and revealed intricate interactions between gender and culture groups. Viewed through the gender lens, the NI group behaved differently from all other immigrant-status groups: whereas women tended to report higher levels of anxiety (and, to a marginal effect, depression) than men in the immigrant groups, the NI group did not show gender differences and even suggested a reverse trend for depression. These effects may be rooted in gender roles – sets of social expectations shaping ‘how men and women should behave’ – thus reflecting cultural assumptions and psychosocial schemata [55]. Traditional gender roles allow for higher ‘emotionality’ among women. In congruence with that, studies of depression and anxiety often report higher levels of both among women, at least when measured using self-report tools [56]. How can we then account for the interaction suggesting that native women and men were the only group that did not show the same pattern? One potential explanation may stem from yet another cultural element – that of the ‘sabra’: native Israeli men and women are often characterized within a culture that values a ‘no nonsense’ attitude, an instrumental approach to challenges, and low regard for emotional expression. Can culture beat gender roles in this case? Future studies may shed light on this phenomenon [57] specifically, as well as on the issue of culture versus gender roles in general as indirectly reflected in my results.

The literature proposes that social support plays a major role in determining wellbeing, and on the reverse side, anxiety and depression [37–39]. Our findings indicate that recent immigrants report lower levels of social support than their veteran peers and others. At the same time social support emerged as a significant factor accounting for depressive symptoms but not anxiety. Unlike most studies in this field, the social support assessed here is actual support rather than reported, perceived support (measured through the number of visits and their lengths during hospitalization). Actual support can work in different paths than perceived support [58] and at times show surprising patterns. For example who provides support to hospitalized older adults (professional workers vs. family member) and what support is offered (emotional vs instrumental) can make a big difference in outcomes [59].

Finally, the results also shed light on the complex nature of the general term of ‘acculturation’. The literature on the psychological processes behind immigration and assimilation describes a non-linear process taking place at multiple levels of cultural assumptions, beliefs, self-identity and self-esteem, to name just a few as well as social identity, interpersonal interactions and identification with the host culture and society. Authors describe multiple paths of negotiating new cultures and identities [60, 61]. Thus future studies may try to examine various paths to acculturation and assimilation of immigrants in a more complex, less uniform manner than this study.

Although the results are compelling, the study’s limitations should be considered when interpreting the results. First, the cultural context of this study is (intentionally) specific. Considering additional and different cultural groups in varying contexts may involve different dynamics and basic assumptions. Second, this study focused on older adults in acute hospitalization settings, where individuals may naturally experience stress and anxiety. While there was an attempt to control for potential intervening factors in the analyses, some potential factors could not be addressed: for example – changes in USSR and Israeli cultures, economics and immigration rules between the 1970s and the 1990, slight changes in human capital and motivation for immigration between all 3 groups of immigrants, are the most prominent ones. As these are mostly dependent on history and maturation effects, there was little that could be done to address these issues and the results are presented within these given contexts. Notwithstanding the importance of studying anxiety in hospitalization settings, it may be of added value for future studies to explore anxiety and stress in more mundane settings, to differentiate state and trait anxiety, and to include a broader selection of cultural backgrounds in the comparison. This study does not account for potential reasons for the differential results comparing anxiety and depression. While the literature suggests that depression may be the result of long-term anxiety (among other factors), long-term studies may shed further light on this issue.

## Conclusions

With its limitations, this study sheds new light on the dynamics of culture and how it may shape anxiety in acutely hospitalized older adults. Culture of origin may play a central role in determining expression of anxiety symptoms and may moderate the role and speed of acculturation. During hospitalization, special attention should be given to the level of anxiety among not only recent but also veteran immigrants. A better understanding of these dynamics may help researchers develop future screening approaches that are more culturally sensitive and effective as well as culture-sensitive interventions to reduce potential disparities in effective health care.
